# Nanotechnological approaches for pentamidine delivery

**DOI:** 10.1007/s13346-022-01127-4

**Published:** 2022-02-25

**Authors:** Ilaria Andreana, Valeria Bincoletto, Paola Milla, Franco Dosio, Barbara Stella, Silvia Arpicco

**Affiliations:** Department of Drug Science and Technology, University of Turin, Via P. Giuria 9, 10125 Turin, Italy

**Keywords:** Pentamidine, Drug delivery, Liposomes, Nanoparticles, Repurposing

## Abstract

Pentamidine (PTM), which is a diamine that is widely known for its antimicrobial activity, is a very interesting drug whose mechanism of action is not fully understood. In recent years, PTM has been proposed as a novel potential drug candidate for the treatment of mental illnesses, myotonic dystrophy, diabetes, and tumors. Nevertheless, the systemic administration of PTM causes severe side effects, especially nephrotoxicity. In order to efficiently deliver PTM and reduce its side effects, several nanosystems that take advantage of the chemical characteristics of PTM, such as the presence of two positively charged amidine groups at physiological pH, have been proposed as useful delivery tools. Polymeric, lipidic, inorganic, and other types of nanocarriers have been reported in the literature for PTM delivery, and they are all in different development phases. The available approaches for the design of PTM nanoparticulate delivery systems are reported in this review, with a particular emphasis on formulation strategies and in vitro/in vivo applications. Furthermore, a critical view of the future developments of nanomedicine for PTM applications, based on recent repurposing studies, is provided.

**Graphical abstract:**

Created with BioRender.com

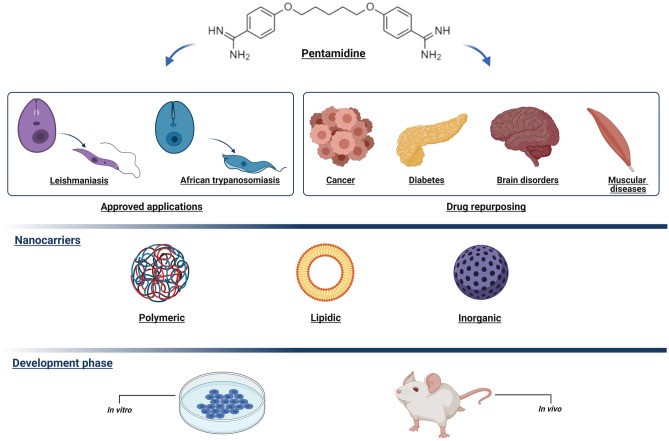

## Introduction

Nanocarrier-based drug delivery has gained ever increasing amounts of attention in recent decades thanks to the characteristics that it can provide: increased drug solubility, modified pharmacokinetics, sustained release, tissue targeting, reduced toxicity, biosafety, and the ability to bypass biological barriers, which result in higher drug efficacy and bioavailability [1–3]. A plethora of different nanocarriers have been proposed for different diseases, such as cancer, central nervous system–related disorders, immune diseases, and, more recently, viral infections [4–7]. Proposed drug nanocarriers differ in their composition (mainly lipid, polymeric, or inorganic) and their structure (matrix or vesicular systems, including those with an aqueous, lipid, or gas core) and several incorporation strategies have been adopted, both for lipophilic and, sometimes, hydrophilic compounds, which is even more challenging [8, 9]. Among the molecules that have been considered for nanoencapsulation, we can find pentamidine (PTM), which is a drug that was initially developed as a synthetic analogue of insulin and is now a Food and Drug Administration (FDA)– and European Medicines Agency (EMA)–approved molecule for the treatment of a range of parasitic infections (Table 1) [10, 11].

**Table 1:** Commercially available products containing pentamidine isethionate

Name	Physical description	Dosage as active ingredient (mg)	Company
Nebupent^®^	Powder for solution	300	Fresenius Kabi, APP Pharmaceuticals
Pentacarinat^®^	Lyophilized powder	200/300	Aventis Pharma, Lepetit
Pentam^®^	Powder for solution	300	Fresenius Kabi, APP Pharmaceuticals
Pneumopent^®^	Lyophilized powder	60	Fisons Pharmaceuticals
Pentamidine isethionate	Powder for solution	200/300	Mayne Pharma, David Bull Laboratories, Taylor Pharmaceuticals, Avet Pharmaceuticals, Seton Pharmaceuticals, Abbott Laboratories

Administration route: by inhalation or i.v. injection

**Fig. 1:**
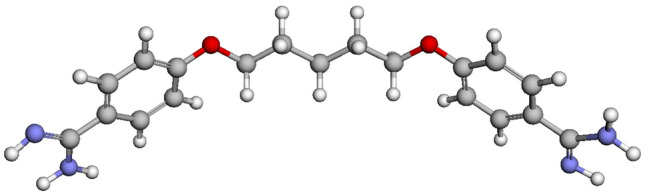
Ball and stick representation of PTM structure (carbon atoms are gray, oxygen red, nitrogen light blue, hydrogen white) [14]

PTM [1,5-bis(4-amidinophenoxy)pentane] (CAS Registry number 100–33-4) (Fig. 1) is an aromatic diamidine, which has been marketed in the form of two salts, isethionate [bis(2-hydroxyethane-1-sulfonic acid)] and mesylate [bis(methanesulfonic acid)], and is administered via the intravenous (i.v.) and intramuscular routes (i.m.) and via inhalation [12, 13]. Its chemical and physical properties indicate that PTM is a solid (melting point 186 °C, decomposes) that is completely water soluble at 25 °C (0.0236 mg/mL, predicted) with a logP of 4 (experimental) and a pKa of 12.13 (predicted) [14]. At physiological pH, both amidine groups are positively charged, meaning that PTM has poor oral bioavailability [15, 16]. Consequently, PTM is used i.v. in the treatment of trypanosomiasis*,* leishmaniasis, and *Pneumocystis pneumonia* (PCP), and is usually inhaled in aerosol form to prevent PCP in high-risk, HIV-infected patients [17–19].

Although PTM is widely used, its application as a curative strategy for infectious diseases and in new therapeutic options urgently requires approaches to improve its therapeutic efficacy, to overcome drug resistance, and to reduce the complications that are caused by its associated adverse effects [20]. The objective of this review is to discuss the advanced strategies that have been adopted to improve PTM delivery. Two different approaches have been developed to overcome the issues inherent in PTM use. The first is the synthesis of new PTM derivatives to provide compounds with better pharmaceutical activity, higher lipophilicity, and lower cytotoxicity. The most promising compounds have been tested in in vitro and in vivo models. This approach is out of the scope of this review. However, papers by Porcheddu et al. and Soeiro et al. provide a full description of the chemical modification methods and synthetic routes used to obtain PTM derivatives [21, 22]. The second approach involves nanotechnology and is the topic of this review. Although not reported in this paper, microcapsules have also been proposed as a means to encapsulate PTM [23–26]; in these preliminary formulation studies, PTM was used as a model drug to analyze the influence of various preparation parameters on the physico-chemical characteristics of drug-loaded poly(lactide-*co*-glycolide) (PLGA) microparticles.

**Fig. 2:**
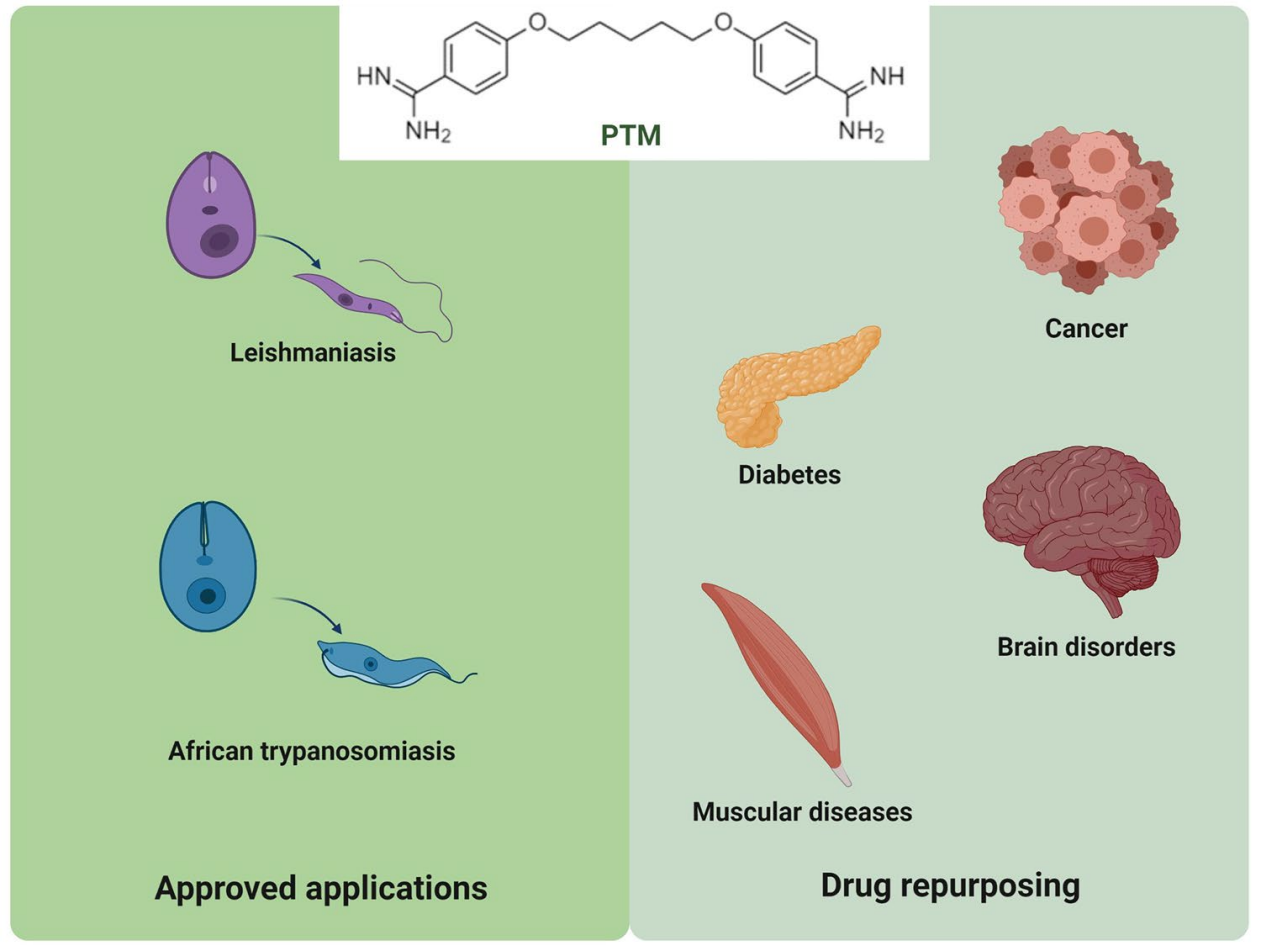
Approved and proposed therapeutic applications of PTM (created with BioRender.com)

Herein, we will initially explore the previously approved and newly proposed therapeutic applications of PTM, focusing on its repurposing in cancer, as well as in brain and muscular disorders (Fig. 2). Secondly, we will consider the most relevant nanomedicine-based strategies that make use of the physico-chemical properties of PTM to overcome its drawbacks and increase its therapeutic compliance. An in-depth analysis of structure, composition, features, benefits, and uses has been conducted for these systems.

## Applications of PTM

### Current PTM applications

PTM was first synthesized in the late 1930s as a hypoglycemic drug against trypanosomes as it was observed that the parasites require glucose for their in vitro maintenance [12]. PTM has been used since the 1940s and is still one of the most frequently used drugs for the treatment of the first stage of human African trypanosomiasis and other neglected diseases, such as malaria and leishmaniasis [27]. Moreover, aerosolized PTM was approved for use in prophylaxis against PCP in HIV-infected patients who are at high risk when infected [28]. It has been reported that the benzamidine moiety is the essential pharmacophore of several commercially available drugs for the treatment of parasitic and fungal diseases; these compounds are characterized by a symmetrical structure in which the aromatic rings are separated by a short acyclic linker [29, 30].

Leishmaniasis and human African trypanosomiasis are infective and endemic diseases that affect the population the world over. Leishmaniasis incidence occurs mainly in three geographical regions (South-East Asia, Latin America, and East Africa) with a total number cases of up to 1.2 million [31, 32]. The parasites are transmitted to humans in the bites of infected female phlebotomine sandflies as a flagellated, metacyclic promastigote, which is phagocyted by host macrophages and then differentiates into non-flagellated, replicative amastigotes, culminating in the expression of clinical disease [33, 34]. Leishmaniasis is characterized by several different clinical manifestations: ulcerative skin lesions that develop at the site of the sandfly bite, multiple non-ulcerative nodules, destructive mucosal inflammation, and disseminated visceral infection [35]*.* The development of the disease is determined by the parasite’s characteristics, vector biology, and host factors, such as immune response [33], and affects organs including the liver and spleen. Pentavalent antimonials, which have a narrow therapeutic index and whose use has largely been limited, are still the first-line drugs in several parts of the world [36]. As a second choice, amphotericin B, PTM, and paromomycin are considered to be curative strategies. Amphotericin B is a highly efficacious antifungal drug, whose associated side effects are ameliorated in its liposomal formulation [37]. Over the years, paromomycin, an aminoglycoside antibiotic, has been used for the topical treatment of leishmaniasis, showing positive results in disease resolution [38]. Many clinically resistant cases have recently been found and treatment failure is an increasing problem. Technological and therapeutic approaches are urgently required if clinical outcomes are to be reached [39, 40]. Although PTM has poor oral bioavailability and pharmacokinetic issues, it can be successfully used for drug-resistant leishmaniasis.

Despite the pharmaceutical drawbacks of PTM, it is also used in the treatment of African trypanosomiasis. Human African trypanosomiasis, also known as sleeping sickness, is considered a neglected disease. It is caused by infection with protozoan parasites that belong to the genus *Trypanosoma*, which is widespread in 23 African countries [41–43]. Trypanosomes are surrounded by a surface glycoprotein that is recognized by the host’s immune system. Nevertheless, a few of the parasites have changed their surface protein to continue proliferating unnoticed by the immune system [44]. It is transmitted via the bite of the blood-sucking tsetse fly, which is a viviparous insect that deposits a fully developed larva, which then needs to become an adult fly and feeds on an infected mammalian host in order to evolve into the infective form. Only about 0–1% of flies carries a mature infection that can be transmitted to another host [45]. The early stage of the pathology is characterized by headaches, weight loss, fever, and lymphadenopathy, which evolve into more serious symptoms that potentially involve the nervous system when the parasite crosses the blood–brain barrier (BBB) [46], producing neurological disturbances including sleep disorder, tremor of the hands, and motor weakness, and can lead to death if untreated [47]. The first-line therapeutic strategy in the early stage currently consists of suramin, melarsoprol, or PTM, although the application of PTM is limited because of its well-known side effects. In particular, hypoglycemia and hyperglycemia are the most common consequences of PTM therapy in patients affected by African trypanosomiasis [41, 48]. Hypoglycemia, with possible progression to insulin-dependent diabetes mellitus, has been reported in HIV-infected patients [49]. Moreover, PTM treatment may be accompanied by a prolongation of the QT interval on electrocardiograms and hypotension [50–52]. Other PTM side effects include abscesses at the site of intramuscular injection, abnormal liver function, pancreatic complications, nephrotoxicity, leucopoenia, thrombocytopenia, and hypocalcaemia [53–56].

### PTM repurposing

As PTM is widely used in clinics and can be readily repurposed, it is the most studied member of the diamidines series, which is a class of compounds that can interfere with many biomolecular targets, meaning that they have been studied as potential drugs for the treatment of a variety of diseases, such as parasitic diseases, tumors, brain disorders (Alzheimer’s and Parkinson’s diseases), hypertension, diabetes, and muscular dystrophy [57]. Indeed, PTM has been investigated, alone and in combination with approved drugs (to foster the discovery of new antimicrobial synergies), in several screenings for the targeting of fungal, Gram-negative, and other pathogens [58–63]. In addition, PTM has recently been identified as a potential blocker of the SARS-CoV-2 3a-channel in a library of 2839 approved-for-human-use drugs, although PTM activity is still to be tested on the whole virus [64].

The mechanism of action of PTM is not well understood despite all of these studies. It has been shown experimentally to interfere with numerous cellular processes [65]. PTM can act as an N-methyl-D-aspartate (NMDA) receptor antagonist, and thus displays neuroprotective effects [66, 67]. It is actively transported into trypanosomes and binds to DNA within the nucleus and kinetoplasts. Additionally, PTM interferes with polyamine synthesis, RNA polymerase activity, enters protozoan cells and binds to transfer RNA, and prevents the synthesis of proteins, nucleic acids, phospholipids, and folate. It is also known to be an antagonist of histone acetyltransferase and calmodulin [68, 69].

Recently, PTM has been reported to exhibit anticancer properties and has shown antiproliferative effects on various human cancer cell types, such as melanoma, prostate, ovarian, colon, breast, lung, and cervical cancers in in vitro and in vivo models. In these studies, several different sites of action were proposed for PTM, but the mechanism of its anticancer activity still remains elusive [70–78].

In another research field, PTM was the first small molecule proposed for the treatment of *Dystrophia myotonica* type 1 (DM1); it gave interesting initial results as it is able to reverse the splicing defects associated with myotonic dystrophy. Indeed, DM1 is a genetic disorder of autosomal dominant inheritance caused by pathological expansions of small DNA sequences ((CTG)_n_) of the DMPK gene. The expanded DNA sequences are transcribed into RNA triplets which aggregate in specific structures called nuclear foci. The unstable structures sequestrate the muscleblind protein (MBNL) in the nucleus, resulting in a local reduction of protein level and characteristic symptoms of DM1 [79]. Several compounds have been shown to bind (CUG)_n_ RNA and release the splicing factors. PTM was selected, together with aminoglycoside antibiotic neomycin B, from a small library of RNA binding compounds using in vitro and in vivo studies. Results showed that PTM is capable of binding the minor groove of AT-rich DNA and of preventing the formation of CUG − MBNL aggregates [80]. Additionally, PTM has recently been indicated as a therapeutic candidate for cardiac defects in DM1 and *Dystrophia myotonica* type 2 using fly heart models (*Drosophila*). Unfortunately, its high toxicity at therapeutic dose in vivo prevented its clinical use [81].

### Administration, fate, and clinical trials of PTM

Because of its toxicity, the pharmacokinetics of PTM has only been studied in patients and has best been described by open, two- or three-compartment models [82, 83]. After the i.v. and i.m. administration of 4 mg/kg, the mean peak serum concentrations were found to be 0.6 mg/L and 0.2 mg/L, respectively, with large volumes of distribution at steady-state of 821 L and 2724 L, respectively. Multiple dosing results in progressive drug accumulation, and the steady-state was achieved after 8 days of i.v. therapy [83]. Mean plasma levels after 21 days of inhaled PTM isethionate at 600 mg/day via a nebulizer averaged 11.8 ± 10 ng/mL in patients with acute PCP [19]. PTM is approximately 70% protein bound and accumulates especially in the liver, spleen, kidneys, and adrenal glands [84]. Biliary excretion is the major elimination pathway for PTM, but release from the liver is slow (99% of the absorbed PTM in the liver is still present 24 h after i.v. infusion), whereas renal elimination only accounted for 4 to 7% of the administered dose [85].

The following clinical trials have been carried out, or are currently ongoing, to investigate the use of PTM for the treatment of a range of diseases.

#### I.v. administration

An i.v. formulation of PTM isethionate, OCZ 103 OS, has been tested as a potential therapy for cancer, including colorectal, pancreatic, and non-small cell lung cancers. However, the development of this formulation appears to have been discontinued after some phase I/II trials were completed (NCT00809796 [86], NCT01378143 [87], NCT00810953 [88], and NCT01844791[89]) (source US National Library of Medicine clinicaltrials.gov).

#### Oral administration

An oral formulation of PTM isethionate is being developed for the treatment of liver diseases, including hepatocellular carcinoma, non-alcoholic steatohepatitis, alcoholic steatohepatitis, non-alcoholic fatty liver disease, and liver metastasis. To date, as far as we know, the oral formulation has only been tested in a phase I study in adult cirrhotic patients with early-stage hepatocellular carcinoma, and safety, pharmacodynamics, and pharmacokinetics data were presented at the 67th Annual Meeting of the American Association for the Study of Liver Diseases [90, 91].

#### Local administration

The topical administration of PTM in a silicone-based gel is currently being tested for the treatment of hypertrophic scars in a phase I clinical trial that is still recruiting (NCT03403621) [92].

Intralesional injections of PTM for the treatment of cutaneous leishmaniasis have been tested in phase II/III studies in combination with topical miltefosine or paromomycin (NCT03445897 and NCT03096457) [93, 94].

## PTM delivery approaches

### PTM for parasitic diseases

**Fig. 3:**
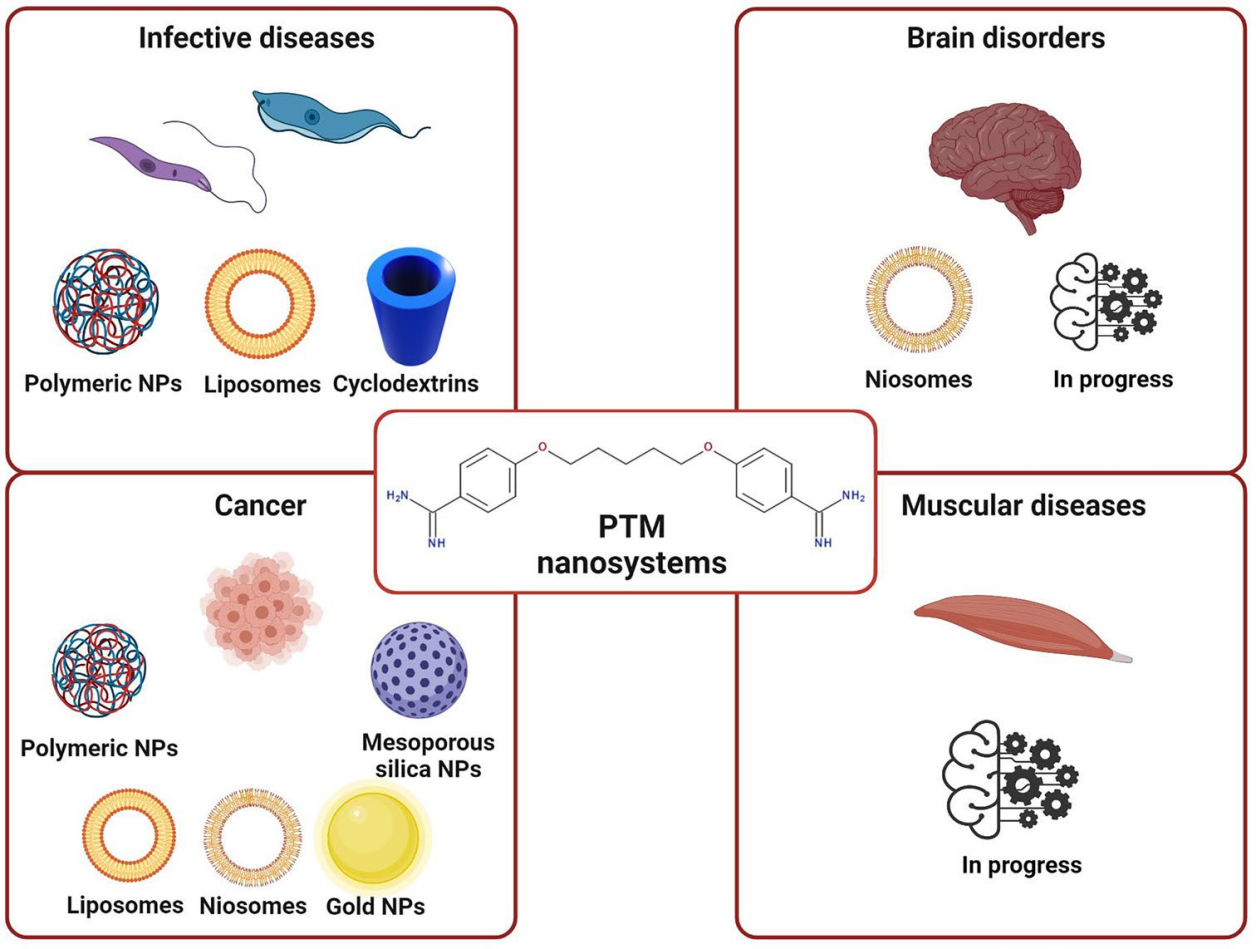
Nanocarriers proposed for the delivery of PTM in different therapeutic approaches (created with BioRender.com)

The formulation of antiprotozoal drugs with nanocarriers is currently highly regarded as a promising approach in the treatment of leishmaniasis and trypanosomiasis [95–97], for which nanocarriers of different matrixes have been demonstrated to be effective [98–100]. The strategies that have been used to encapsulate PTM with drug delivery systems for the treatment of parasitic diseases are discussed below, and the characteristics of the PTM-based formulations are summarized in Table 2 and Fig. 3.

**Table 2:** Recapitulative table of the described nanosystems that encapsulate PTM

Class of nanocarrier	Nanocarrier composition	PTM form	Preparation technique	Development phase	Admin. route	Pathology	Ref
**Lipidic**	Mannose-grafted liposomes	Isethionate salt	Thin lipid evaporation and hydration method	In vivo leishmaniasis hamster model	s.c	Leishmaniasis	Banerjee et al. [105]
	PEG-coated liposomes	Free base or isethionate salt	Thin lipid evaporation and hydration method or transmembrane gradient	In vitro test on a cancer cell line	/	Cancer	Stella et al. [148]
	PEG-coated liposomes	Isethionate salt	Thin lipid evaporation and hydration method	In vivo cancer mouse models	i.v	Cancer	Merian et al. [149]
**Polymeric**	Polymethacrylate	Isethionate salt	Emulsion polymerization	In vivo	i.v	Leishmaniasis	Paul et al. [112]
				BALB/c			
	Methacrylate	Methane sulfonate solution	Emulsion polymerization	In vivo	i.v	Leishmaniasis	Durand et al. [115]
				BALB/c			
	PCL	Isethionate salt	Double solvent evaporation	In vitro brain endothelial cells	/	Leishmaniasis	Omarch et al. [116]
	PEG-chitosan	Isethionate salt	Coacervation method	In vivo *T. brucei* mouse model	i.p	Trypanosomiasis	Unciti-Broceta et al. [119]
	PLA	Free base	Nanoprecipitation	In vivo BALB/c	i.v	Leishmaniasis	Paul et al. [112, 123] and Durand et al. [125]
	PEG-PLGA-PTM bioconjugate	Isethionate salt	Water/oil emulsion method	In vitro *L. infantum* amastigote-infected macrophages	/	Leishmaniasis	Scala et al. [127]
	PEG-PLGA	Not specified	Water-in-oil-in-water double emulsion/solvent evaporation	In vivo African trypanosomiasis mouse model	i.p	Trypanosomiasis	Arias et al. [128]
	PLGA	Free base	Water-in-oil-in-water double emulsion/solvent evaporation	In vivo leishmaniasis mouse model	s.c./os	Leishmaniasis	Valle et al. [129]
	Cyclodextrins	Isethionate salt	/	In vivo leishmaniasis mouse model	i.v./os	Leishmaniasis	De Paula et al. [134]
	Hyaluronic acid and polyarginine	Isethionate salt	Polyelectrolyte complexation	In vitro tests on cancer cell lines	/	Cancer	Carton et al. [147]
	PLGA and PEG-PLGA	Free base	Nanoprecipitation	In vitro test on a cancer cell line	/	Cancer	Stella et al. [148]
	Polysorbate 20, cholesterol, dicetyl phosphate, chitosan (niosomes)	Isethionate salt	Thin film hydration method	Physico-chemical characterization	/	Alzheimer’s disease	Rinaldi et al. [141]
	Polysorbate 20, cholesterol, dicetyl phosphate, chitosan (niosomes)	Isethionate salt	Thin film hydration method	In vivo Parkinson’s disease mouse model	Nasal	Parkinson’s disease	Rinaldi et a. [143]
	Polysorbate 20, cholesterol, dicetyl phosphate, chitosan (niosomes)	Isethionate salt	Thin film hydration method	In vitro tests on human biopsies	/	Cancer	Seguella et al. [145]
**Inorganic**	Functionalized mesoporous silica	Free base or isethionate salt	Sol–gel method and functionalization	Physico-chemical characterization	/	/	Peretti et al. [151]
	PEG-gold	Not specified	Seed-mediated growth and PEGylation	In vitro tests on cancer cell lines	/	Cancer	Her at al. [153]

#### Liposomes

Liposomes, as drug delivery systems, are vesicles composed of phospholipids organized in concentric bilayers that enclose one or more aqueous spaces. A description of the preparation methods and their characteristics can be found elsewhere [101, 102]. The advantage of using liposomes as vehicles is their rapid accumulation in phagocytic cells as they are recognized as foreign particles, allowing liposomes to enhance the non-specific host defense [103]. However, there are only a few studies on the encapsulation of PTM in liposomes for use against *Leishmania* and human African trypanosomiasis. Nevertheless, after several different in vitro tests, researchers have found that glycoside-bearing liposomes can be used as systems to deliver drugs to macrophages in vivo [104]*.* On this basis, Banerjee et al. have described how sugar-grafted liposomes were prepared and tested against leishmaniasis in hamsters*.* Golden hamsters were infected via the intracardial passage of strain AG 83 from an Indian kala-azar patient. Different types of sugars, including mannose, galactose, and glucose, were tested. These formulations were injected subcutaneously (s.c.) into each animal every 3 days for a total of four doses over 10 days. Mannose-grafted liposomes that encapsulated PTM isethionate reduced the parasite load of the spleen by 85.1%, whereas liposomes without sugar lowered it by only 46.6%. Consequently, mannose-grafted liposomes were found to be the most efficient PTM delivery system, compared to other sugar-grafted carriers and sugar-free vesicles [105].

In another study, PTM isethionate was loaded into liposomes and tested against *Acanthamoeba*, which is an opportunistic protozoan pathogen that can cause blinding keratitis and fatal granulomatous encephalitis. Liposomes were prepared via the dehydration-rehydration method using L-α-phosphatidylcholine and either cholesterol or ergosterol. The results showed that both liposomal cholesterol-PTM and ergosterol-PTM were about 10 times more effective than the free drug at preventing the binding of *Acanthamoeba* to human brain microvascular endothelial cells. Moreover, loaded liposomes were more effective at reducing parasite-mediated human cell cytopathogenicity than free PTM [106].

#### Polymeric nanoparticles

The use of polymers has been widely investigated as they offer several advantages, such as controlled drug release, the protection of drugs against premature degradation, and surface engineering [107, 108]. Studies on PTM delivery have reported the applicability of polymeric nanoparticles as potential tools for the improvement of the drug’s pharmacokinetics and pharmacodynamics drawbacks [109]. In one of the first studies on the encapsulation of anti-*Leishmania* therapeutic agents into polymeric nanoparticles, Gaspar et al. described the ability of polyalkylcyanoacrylate nanoparticles to reduce the effective dose (ED_50_) of primaquine. Increased effectiveness was demonstrated in vitro in macrophages infected with the *Leishmania* parasite, compared to free primaquine [110]. The emerging knowledge of nanomedicine and the potential applicability of PTM have led to the same technological approach being studied and activity investigations being performed on the formulation of PTM with different acrylate nanoparticles. Optimized formulations of PTM-loaded methacrylate nanoparticles (mean size: 350 nm) were firstly tested in vitro using a strand of *Leishmania major* MON 25 and monocytes U 937, with increased drug efficacy being reported, compared to the free drug [111]. These interesting results led to Fusai et al. describing PTM-loaded polymethacrylate nanoparticles and their efficacy in vivo. The nanoparticles were first prepared via the emulsion polymerization technique using PTM isethionate, based on electrostatic interactions between drug and polymer [112]. Subsequently, in vivo studies on BALB/c inbred mice were performed to confirm the superior effectiveness of the PTM-loaded nanoparticles. The mice that were administered the free drug by i.v immediately showed side effects, while in the group that received drug-loaded nanoparticles there was no evidence of shock after the injection. The state of infection was evaluated after 21 days by counting the number of amastigotes in the liver. Seventy-seven percent parasite suppression was reported in the group treated with loaded nanoparticles relative to the control group [113, 114]. Due to the increased interest in polyacrylate nanoparticles, Durand et al. have tested the activity of PTM-loaded methacrylate nanoparticles on a *Leishmania infantum* mouse model. The efficacy of encapsulated PTM against a different form of *Leishmania* was confirmed by the low value of ED_50_ obtained, demonstrating the ability of methacrylate nanoparticles to increase the efficacy of PTM and reduce side effects [115]. Nevertheless, the main problem of acrylate nanoparticles is their low biodegradability, which can be inconvenient for in vivo administration.

Moving on to other polymers, recent studies have involved the use of polycaprolactone polymer (PCL) loaded with PTM to enhance permeation through the BBB. PTM-loaded PCL nanoparticles were obtained using the double solvent evaporation method and their physiochemical characteristics were evaluated. Their spherical shape decreased from 345 to 270 nm after complexation with PTM, with a negative zeta potential value of around −30 mV and 12% drug release over a period of 24 h. The integrity of the BBB was evaluated in vitro on brain endothelial cells after loaded and unloaded nanoparticles were added; no significant tight junction alterations were observed, while loaded nanoparticles were shown to transport 66% of the PTM across the BBB [116].

Drug resistance, which complicates the management of *Leishmania* and African trypanosomiasis, is an important issue in the treatment of parasitic diseases [117, 118]. African trypanosome is generally covered by a single variant surface glycoprotein, termed VSG [42]. Combining PTM with targeted drug delivery systems overcomes possible drug resistance by taking advantage of the delivery systems’ enhanced absorption into target cells, which is caused by surface engineering. Based on the chemical and biological structure of the infective parasite, Unciti-Broceta et al. have developed PEGylated chitosan nanoparticles that are surface-targeted with nanobodies that are able to specifically recognize specific VSG regions on a parasitic surface [119]. Indeed, these nanobodies are small enough to be able to infiltrate the dense parasite VSG coat and specifically recognize conserved epitopes common to the majority of the VSG molecules [120]. To this aim, nanobodies were coupled to the PEGylated PLGA nanoparticles via the EDC/NHS chemistry. Nanoparticles were obtained using coacervation methods and by selecting the right PEG chain length to allow the nanobodies to bind onto the parasitic membrane. Spherical-shape nanoparticles had a size of around 135 nm and a negative zeta potential value for both loaded and blank nanoparticles, meaning that PTM was completely loaded inside chitosan nanoparticles. In vitro drug release had a pH-depend profile that increased in an acidic environment due to the chemical properties of PTM, which is advantageous for delivery to the parasitophorous vacuole of parasites (pH 5). The in vitro tests on the PTM-loaded nanoparticles showed a reduction in inhibitory concentration, compared to the unloaded and untargeted formulations. The in vivo therapeutic efficacy was evaluated in a mouse model infected by *Trypanosomiasis brucei* in order to provide further confirmation of the in vitro results. Mice were treated by intraperitoneal injection (i.p.) with PTM-loaded targeted nanoparticles, and the complete resolution of infection was observed, unlike the group receiving the same nanoparticle without the nanobody surface functionalization. The possibility of the alternative internalization of an active agent may decrease spontaneous drug resistance that can limit or reduce the applicability of some antiprotozoal drugs [119].

Other nanomedicine approaches have focused on poly(D,L-lactide) (PLA) and PLGA polyesters as they are known to be biodegradable and biocompatible [121, 122]. Paul et al. have described the preparation, the physiochemical properties and the stability of PTM-loaded PLA nanoparticles. Loaded nanocarriers were obtained via the nanoprecipitation technique using a combination of phospholipids and a poloxamer, which influences the physiochemical characteristics of the nanosuspension. PTM loading improved when the lipid content was directly increased: indeed, varying the phospholipid concentration from 0.6 to 1.25% (w:v), the highest drug loading (75.8%) was obtained with the highest concentration of phospholipids (1.25%). The formulation was stable over 9 months, in terms of its size and morphology [123, 124]. The biological evaluation of PTM-loaded PLA nanoparticles was performed by determining the ED_50_ against *Leishmania infantum* in male adult BALB/c mice by i.v. injection. A comparison between free PTM and loaded PTM showed the ability of PLA nanoparticles to induce a threefold increase in PTM activity (ED_50_: 1.05 mg/kg for PTM vs. 0.32 mg/kg for loaded nanoparticles) [125].

Due to the growing interest in polymeric nanoparticles for antiprotozoal drug delivery, PLGA nanocarriers have been widely described. In particular, investigations into PLGA nanoparticles as a potential tool to deliver PTM have been reported. Starting from the formulation of PLGA microparticles to improve the pharmacokinetics and pharmacodynamics drawbacks of PTM [126], several research groups have focused their attention on nano-structured PLGA drug delivery systems. Recently, Scala et al. have proposed the covalent linkage of PTM with a PEG-PLGA block copolymer and a hyaluronic acid backbone by click chemistry. They aimed to evaluate the activity of free PTM and PTM bioconjugates. Moreover, they compared the therapeutic effects of PTM-loaded PEG-PLGA-PTM nanoparticles. The anti-leishmanial activity of PTM bioconjugates and PTM nanoparticles was assessed in vitro against infected macrophages. PTM bioconjugate and PTM nanoparticles displayed an IC_50_ 3.6 and 3.4 times lower than free PTM, respectively. In particular, the bioconjugate with hyaluronic acid was found to be the most active derivate, characterized by an IC_50_ value of 1.7 µM compared to 7.5 µM of free PTM, confirming the effectiveness of the hyaluronic acid targeting scaffold [127].

**Fig. 4:**
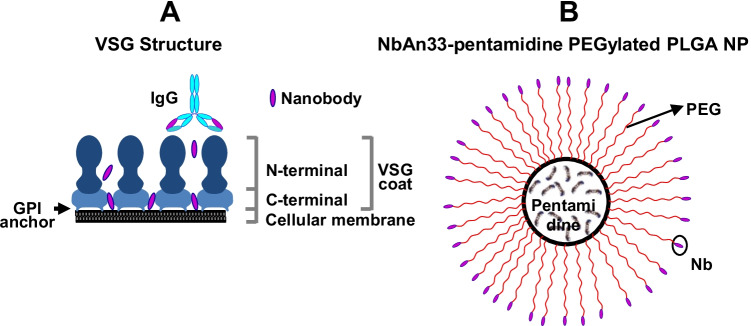
**A** Schema of the VSG coat on trypanosome surface, in which the NbAn33 nanobody recognizes a hidden conserved epitope within the GPI anchor. **B** Schema of a NbAn33-PTM-PLGA nanoparticle. Reproduced with permission from [128]. Copyright Elsevier 2015

As has already been reported for polyacrylates, PLGA nanoparticles were loaded with PTM and surface-engineered to efficiently recognize the surface of the protozoan pathogen *Trypanosoma brucei*, in particular a hidden conserved epitope within the glycophosphatidylinositol (GPI) anchor of the VSG protein (Fig. 4). PEGylated PTM-loaded PLGA nanoparticles were prepared via the water-in-oil-in-water (w/o/w) double emulsion/solvent evaporation technique, followed by PLGA functionalization with PEG chains and NbAn33 nanobodies (both through the EDC/NHS approach) for protozoan-surface recognition. A monodispersed nanosuspension that was characterized by a 145 nm mean diameter and a negative zeta potential value, of around -20 mV, was produced. In vitro studies highlighted the threefold increased efficacy of PTM-loaded PLGA nanocarriers. Moreover, the efficacy of the polymeric formulation increased fourfold again after surface functionalization by nanobodies, which underlines the importance of active targeting in enhancing cellular uptake in parasitic diseases. In vivo tests in an acute mouse model of African trypanosomiasis confirmed the urgent need for surface-nanobody conjugation. The infections of mice injected i.p. with the nanobody-targeted formulation were completely resolved at a tenfold lower dose. In vivo PTM activity was significantly optimized by PLGA encapsulation and, most of all, by surface functionalization, demonstrating that the trypanosome surface was an excellent therapeutic target [128].

PTM-loaded PLGA nanoparticles were developed, using the same preparation technique, for oral delivery in the treatment of *Leishmania* [129]. For the first time, the application of nanoparticles was devoted to the oral delivery of an antiprotozoal drug. The formulation of PTM-loaded PLGA nanoparticles included polyvinyl alcohol (PVA) as a stabilizer, which reduced the zeta potential value to around neutrality and gave a size of around 250 nm. The in vitro release profile confirmed the data reported by Arias et al. which described an initial burst release followed by constant release over 25 days [128]. In this study, the evaluation of the possible oral delivery of PTM-loaded nanoparticles was innovative and highly interesting. In vivo tests performed in a murine visceral leishmaniasis model aimed to compare infection resolution via the oral and s.c. delivery of loaded nanoparticles. S.c. administration did not lead to significant reductions in spleen and liver weight, which is the major end point in infective-disease studies. Interestingly, a reduction in the parasite presence in the spleen occurred after oral administration, which demonstrated the enhanced PTM uptake that was achieved by the oral route [129].

An innovative approach has recently been proposed to understand the toxicity of PTM through the BBB in the treatment of African trypanosomiasis. Sanderson et al. have evaluated the complexation of PTM with Pluronic, a polymer that can act as a stabilizer to prevent aggregation, opsonization, and recognition by macrophages and thus increase drug half-life, but that can also assemble into micelles to encapsulate drugs. Although the study did not show the increased uptake of PTM by the brain, this is, nonetheless, a new formulation of PTM that has been proposed as an alternative approach to test the activity of PTM on an African trypanosomiasis model [54].

#### Inclusion complexes

Cyclodextrins (CD) are composed of 6, 7, or 8 glucopyranose units (named α-, β-, and γ-CD, respectively) linked by a glycosidic bond and organized in a truncated cone. Polar hydroxyl groups on the molecule’s border confer water solubility, while the inner hydrophobic cavity allows a variety of drugs to be hosted, forming inclusion complexes. β-CD are useful as they can improve the physico-chemical and biological properties of the included molecules, increasing tolerance and alleviating the drawbacks [130–133].

De Paula et al. have investigated the inclusion of PTM into the β-CD cavity, and then studied its activity when given via oral administration. First, β-CD that contained PTM (β-CD:PTM) were prepared using a freeze-drying method and tested in vivo against *Leishmania infantum chagasi* parasites. Groups of mice were infected in the tail vein with promastigotes and treated with free PTM, β-CD:PTM (1:1), a saline solution, and Glucantime® (meglumine antimoniate, an approved antiprotozoal drug). No differences were found in the groups treated with free PTM and the control group, which was treated with the saline solution, in the liver and spleen. Orally administered β-CD:PTM and Glucantime® had similar efficacy, which was in contrast with the results obtained with orally administered PTM, which did not induce a significant reduction of the parasite burden. In addition, β-CD:PTM showed a greater reduction of parasitemia in the liver. The results demonstrated that β-CD:PTM are a promising alternative for the treatment of leishmaniasis [134].

### PTM for other therapeutic purposes

As reported above, PTM has also been considered for possible clinical repositioning as an anti-Alzheimer’s, anti-Parkinson’s, and anticancer drug. PTM has been incorporated into different nanocarrier systems, whose composition and physico-chemical characteristics depend on both the administration route and physiopathological features, for each of these diseases (Table 2 and Fig. 3).

#### Niosomes

Besides liposomes, other nanoscale vesicles that contain aqueous cores have been used to deliver PTM, namely niosomes. Also known as non-ionic surfactant vesicles, niosomes are self-assembled vesicular nanocarriers formed of one or more layers of non-ionic surfactants and additives; niosomes can overcome some of the problems associated with liposomes, such as their low physical stability and high manufacturing costs [135].

PTM-loaded niosomes have been designed to reach the brain via intranasal administration, bypassing the BBB as a possible tool in brain disorders. Indeed, PTM inhibits S100 calcium-binding protein B (S100B) in glial cells through the blockage of the interaction between the S100B and p53 proteins [136]; PTM is able to exert anti-inflammatory and neuroprotective effects [137], as high expression levels of S100B have been shown to cause neuroinflammation and the progression of brain diseases [138, 139]. Nevertheless, PTM has a poor capacity to cross the BBB and reach the brain after intranasal administration [140]. In a first approach to the delivery of PTM to the brain, the drug was associated to niosomes for the treatment of Alzheimer’s disease [141]. Commercial PTM isethionate was located in the bilayer, with an entrapment efficiency of about 10%, using the thin film hydration method. To overcome the low drug availability that is caused by rapid mucus clearance in the nasal cavity, mucoadhesive properties were provided to PTM-loaded niosomes by coating them with chitosan glutamate (CG) thanks to the electrostatic interactions between positively charged chitosan and the negatively charged dicetyl phosphate on the niosome surface. The interaction with mucin was confirmed using dynamic light scattering and fluorescence turbidity measurements for both uncoated and CG-coated niosomes, and is probably due to a non-specific mucin interaction. Nevertheless, CG can also act as a stabilizer and a penetration enhancer by widening the tight junctions between mucosal epithelial cells [142].

Further studies with PTM-niosomes were conducted in a Parkinson’s disease murine model. In particular, chitosan-coated niosomes that contained PTM (entrapment efficiency of about 25%) were administered daily via the intranasal route in subchronic (1-methyl-4-phenyl-1,2,3,6-tetrahydropyridine) hydrochloride (MPTP)–intoxicated mice. The results showed that PTM-niosomes allowed 35–40% of the drug to be delivered into the brain. Moreover, the intranasal administration of PTM-loaded niosomes leads to an improvement of all the disease hallmarks and a significant reduction in neuroinflammation markers in mice, leading to a significant improvement in parkinsonian motor dysfunctions [143].

The S100B protein is also constitutionally expressed by enteric glial cells in the enteric nervous system; when it is overexpressed, it has a role in the perpetuation of a tumor-promoting microenvironment, driving the progression from chronic intestinal inflammation to colonic neoplastic lesions [144]. Thus, in order to evaluate PTM as a potential anticancer drug, the expression levels of the S100B and p53 proteins were quantified in human biopsies that were derived from controls, peritumoral tissues, ulcerative colitis, and colon cancer patients, at baseline and after the addition of PTM-loaded niosomes. The results showed that encapsulated PTM inhibits S100B activity and rescues p53 expression, leading to pro-apoptotic control in colon cancer. Moreover, the combination of PTM with gastro-resistant and bioadhesive niosomes will allow the drug to increase colonic bioavailability after oral administration [145].

#### Polymer nanoparticles and liposomes

**Fig. 5:**
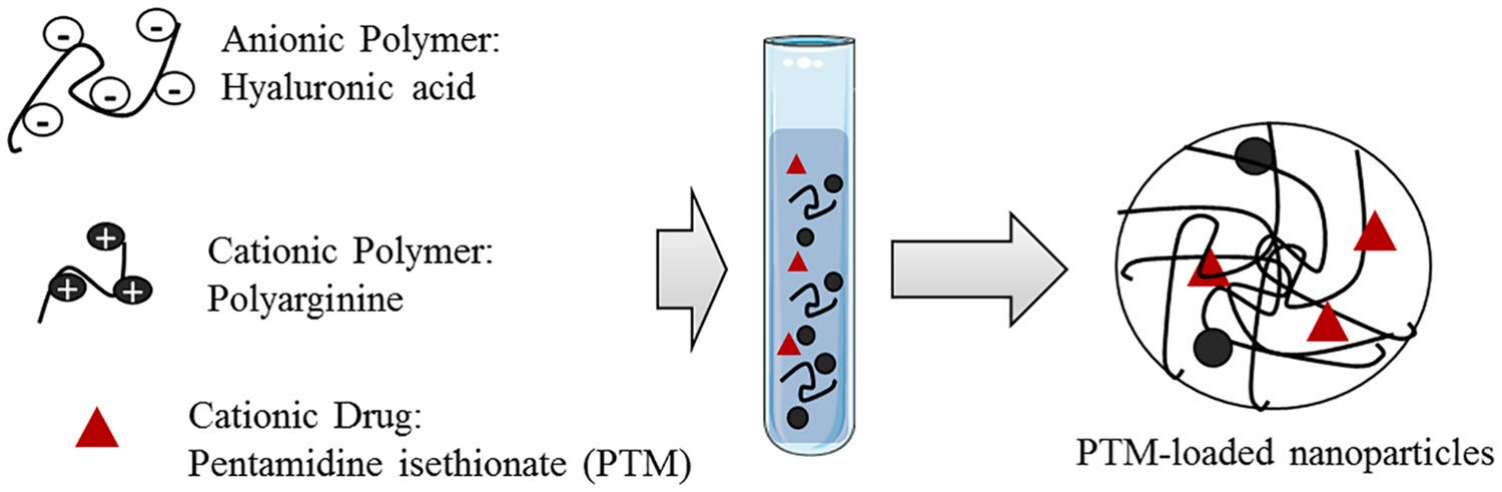
Formation of PTM-loaded nanoparticles by polyelectrolyte association. Reproduced with permission from [147]. Copyright Elsevier 2019

In other approaches, the design of biocompatible PTM-loaded polymer nanocarriers has taken advantage of the drug’s chemical structure, as PTM has two terminal amidine groups that are protonated in a wide pH range, including physiological/neutral conditions [146]. For example, PTM has been combined with nanocomplexes, made of the polymers hyaluronic acid and polyarginine, thanks to the electrostatic interactions between the anionic polysaccharide and the cationic drug (Fig. 5) [147]. In these structures, polyarginine was used to crosslink hyaluronic acid and form nanoparticles.

The measurements of the counterion isethionate released from the drug during nanoparticle formation, using ion exchange chromatography, showed a high drug encapsulation efficiency (80%). A comparison of the in vitro anticancer activity of free and encapsulated PTM in human lung (A549) and breast (MDA-MB-231) cancer cell lines suggested that the polyelectrolyte complexes allow enhanced cell internalization of the cationic drug to occur, with this probably being due to its more hydrophobic character after complexation, and increased cytotoxicity was thus observed. Moreover, both cell lines express CD44 receptors that specifically recognize hyaluronic acid. The polyelectrolyte complexes can therefore be selectively targeted towards these cancer cells through the interaction with these receptors [147].

Other biocompatible polymers have been used to deliver PTM to cancer cells via electrostatic interactions. In particular, negatively charged PLGA (with and without PEG) has been used to load the PTM free base (obtained from the commercial PTM isethionate to increase the drug lipophilicity) via the nanoprecipitation technique. In the same study, PTM was encapsulated into liposomes, as the free base or with different counterions, namely PTM citrate and PTM sulfate, for comparison. PTM base–containing liposomes were prepared using the thin lipid film hydration method by mixing phospholipids, cholesterol, and the PTM base in the organic phase, while PTM citrate and PTM sulfate were encapsulated into liposomes with a transmembrane citrate or sulfate gradient, starting from PTM isethionate in the external liposomal phase. This approach increased the encapsulation efficiency from 20% for the PTM base to 30% for PTM citrate and 45% for PTM sulfate. In PLGA nanoparticles, the PTM free base was almost totally incorporated (about 90%) thanks to the lipophilic and ionic interactions between PLGA and PTM. Drug release was faster for polymer nanoparticles than liposomes and, as a consequence, the cytotoxicity (evaluated in the A2780 human ovarian carcinoma cell line) was different and tunable according to the nanocarrier and the PTM form [148].

Moving on to liposomes in the anticancer field, other liposomal PTM formulations have been prepared using saturated/unsaturated phospholipids of different chain lengths and cholesterol content, with or without PEG on the outer surface. In this study, PTM isethionate was incorporated, using the thin lipid film hydration method, via its addition to the aqueous phase in the hydration procedure. The liposome composition greatly influenced the particle mean size, zeta potential, and encapsulation efficiency, which was in the 1–13% range. The pharmacokinetics profile of PTM, in five liposome formulations administered in non-tumor-bearing mice, showed that both the clearance and volume of distribution of PTM were significantly lower when administered in liposomes, compared to the free drug. PTM biodistribution and tumor accumulation after i.v. administration were evaluated in tumor-bearing mice (colorectal, lung, ovarian, and breast cancer). The results showed that liposomal PTM increased tumor drug accumulation and lowered drug exposure to vulnerable tissues, such as the kidneys, compared to the free drug [149].

#### Inorganic nanoparticles

Inorganic nanocarriers have also been used, with functionalized mesoporous silica nanoparticles (MSN) having been proposed for PTM delivery. MSN are versatile and stable materials with a mesoporous structure, a high specific surface area, and a huge, tunable pore volume that can accommodate large amounts of bioactive compounds. Both the external and inner surfaces can be functionalized with linkers, and this functionalization can affect therapeutic agent loading and release profiles and greatly influence MSN fate in biological fluids [150]. In particular, the encapsulation of PTM isethionate and the PTM free base into bare (MSN-OH), aminopropyl (MSN-NH_2_), cyanopropyl (MSN-CN), and carboxypropyl (MSN-COOH)–functionalized MSN has been investigated. PTM isethionate was not incorporated into any MSN, while the PTM free base displayed significant drug loading with MSN-OH and MSN-COOH thanks to the electrostatic interactions. MSN-CN showed slower drug loading, while the adsorption capacity of MSN-NH_2_ was found to be negligible. The presence of different functional groups also influenced the release of the drug: MSN-COOH showed higher retention than MSN-OH, indicating that the hydrophobic interactions of the drug with the grafted propyl chains had a significant impact on the stabilization of the host–guest complex [151]. This work allowed the authors to select MSN-COOH as the most promising functionalized MSN to be further used as drug delivery system that could be proposed for PTM delivery towards either cancer or muscle cells [152].

PTM has also been considered for use in combination with PEG-stabilized gold nanoparticles (AuNP) to enhance the effect of radiotherapy, in two triple-negative breast cancer cell lines, by targeting multiple pathways of radiosensitization. Although the loading of PTM onto AuNP was not the goal of the study, the authors evaluated the effect of AuNP as a single agent, in combination with PTM and after the pre-adsorption of the drug onto the nanoparticle surface to elucidate the underlying mechanisms of radiosensitization by the AuNP-PTM combination. The results showed that PTM enhances the radiosensitization effect of PEG-AuNP by exerting a dual action: by inhibiting radiation-induced DNA repair and increasing the cellular uptake of PEG-AuNP after surface adsorption, causing a change in the particle surface charge [153].

## Conclusion

**Fig. 6:**
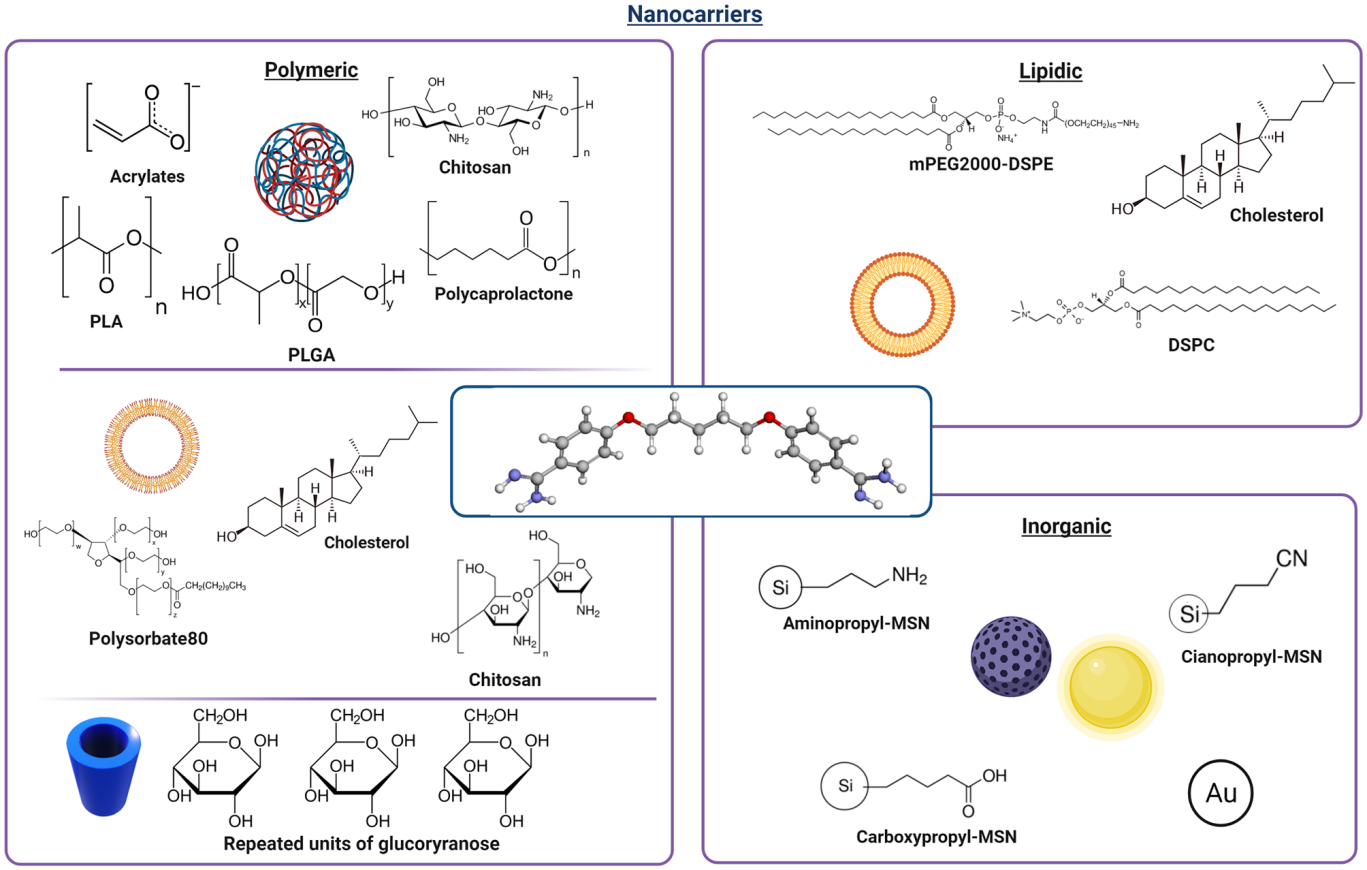
Summary of PTM-loaded nanocarriers as a function of their chemical composition (created with BioRender.com)

PTM is an old, FDA- and EMA-approved drug whose repurposing and delivery via nanocarriers of different composition have been extensively investigated (Fig. 6). Indeed, the interest in PTM is due to its well-known toxicology profile and multifactorial mechanism of action, although the latter has not yet been fully understood. PTM is appealing in terms of the design of nano-sized carriers as it is a small molecule whose physico-chemical characteristics (e.g., solubility in water and organic solvents) can be changed according to the PTM form (salt or free base) and the counterion in the salts. Moreover, PTM is positively charged at physiological pH, thus allowing both electrostatic and lipophilic interactions with nanocarrier components that can, in this way, reduce the positive charge of the drug and increase its passage through the cell membrane. The incorporation into nanocarriers may also modify the toxicological profile of PTM and/or allow the drug to be actively targeted towards the target site, lowering the administered dose. Finally, the reported variety of nanocarriers that are suitable for PTM delivery facilitate the tuning of drug encapsulation efficiency and release rate as a function of the disease and patient features. This aspect is of a crucial importance, as it has been demonstrated that PTM could be repurposed for pathologies other than infectious ones, especially rare muscular diseases, and this aspect justifies a renewed interest in PTM and further multidisciplinary efforts for investigations in this field.

However, several papers on PTM-loaded nanocarriers reported in the present review describe only preliminary in vitro results or even just formulative studies. Therefore, a deeper analysis on the in vivo behavior of PTM-loaded nanocarriers is needed, particularly using models with animals infected with parasites or affected by the diseases for which PTM has been repurposed.

In conlusion, it is worth adding that the combination of PTM and nanocarriers implies also well-known critical features, especially in terms of industrial feasibility, as the system complexity increases. Indeed, the encapsulation of PTM would involve higher costs if compared to the production of the free drug. Furthermore, problems concerning the scalability, reproducibility (in terms of physico-chemical characteristics, such as mean size, zeta potential, drug loading), and compliance to regulatory standards can hamper its use in the clinic. All these issues have to be taken into account and evaluated as a function of the repurposed application.

## Data Availability

Not applicable.
